# Can medicines development improve outcomes in asthma and chronic obstructive pulmonary disease management by driving effectiveness?

**DOI:** 10.1186/s12931-019-1127-6

**Published:** 2019-08-02

**Authors:** David A. Leather, Louisa Yates, Henrik Svedsater, Loretta Jacques, Susan Collier, Danielle Powell, Rupert Jones

**Affiliations:** 1 Global Respiratory Franchise, GlaxoSmithKline plc., Brentford, Middlesex UK; 2 Value Evidence & Outcomes, GlaxoSmithKline plc., Brentford, Middlesex UK; 3 Clinical Sciences, GlaxoSmithKline plc., Uxbridge, Middlesex UK; 4 UK Medical, GlaxoSmithKline plc., Uxbridge, Middlesex UK; 5 Community and Primary Health Care, Faculty of Medicine and Dentistry, Plymouth University, Plymouth, UK

**Keywords:** Asthma, Chronic obstructive pulmonary disease (COPD), Disease management, Effectiveness, Medicines development, Outcomes, Respiratory, Salford Lung Studies

## Abstract

Despite the availability of treatment guidelines and inhaled medications for asthma and chronic obstructive pulmonary disease (COPD), much remains to be done to lessen the burden of these respiratory diseases for patients. The challenge of selecting effective and efficacious drugs for patients is a key focus area for healthcare professionals. Here we discuss the concept of “drivers of effectiveness”— features of a medicine which may increase or decrease its effectiveness in the presence of real-world factors — and highlight the importance of considering these drivers in the early stages of drug development, and exploring their impact in carefully designed pragmatic trials. Using the Salford Lung Studies (SLS) in asthma and COPD as an illustrative example, we discuss various features of the inhaled corticosteroid/long-acting β_2_-agonist combination, fluticasone furoate/vilanterol (FF/VI), as potential drivers of effectiveness that may have contributed to the improved patient outcomes observed with initiation of FF/VI versus continuation of usual care in the UK primary care setting.

## Background

The worldwide burden of asthma and chronic obstructive pulmonary disease (COPD) remains high. The global state of progress in improving health outcomes for patients with asthma has largely plateaued and there has been little advancement towards helping a large proportion of patients whose asthma remains uncontrolled [1, 2]. Similarly, COPD continues to be associated with high morbidity [3, 4] and according to 2016 World Health Organization estimates, COPD was the third leading cause of mortality worldwide [5]. Guidelines for the management of asthma and COPD have existed for, and evolved over, many decades. Likewise, effective medicines for asthma and COPD have been available for many years. Highly controlled efficacy studies, for example the Gaining Optimal Asthma ControL (GOAL) study [6], have demonstrated that good asthma control is possible in the majority of patients. Despite these evidence-based guidelines and medicines with proven efficacy in highly controlled clinical trials, we appear to be failing to make the headway we might expect in lessening the burden of respiratory diseases for patients.

The reasons for poor asthma control and lack of progress in asthma care have been widely described [1, 2, 7–10]. Haughney et al. [10] have defined some of the obstacles to achieving good asthma control (Table 1). Similar barriers have been described for COPD [3, 11].

**Table 1:** Obstacles to achieving good asthma control

• Wrong diagnosis	
• Incorrect choice of inhaler or poor technique	
• Lifestyle choices (e.g. smoking)	
• Co-morbidities (e.g. rhinitis, obesity)	
• Individual variation in response to treatment	
• Patient beliefs and adherence	

While there is a strong evidence base supporting the efficacy of currently available medicines for asthma and COPD, their prescription by clinicians and use by patients is suboptimal and leaves many patients at risk due to poor disease control. Incorrectly prescribed and poorly utilized treatments are also costly and lead to inefficiency in healthcare systems. The challenge of selecting effective and efficacious drugs for patients is a key focus area for healthcare professionals.

**Fig. 1:**
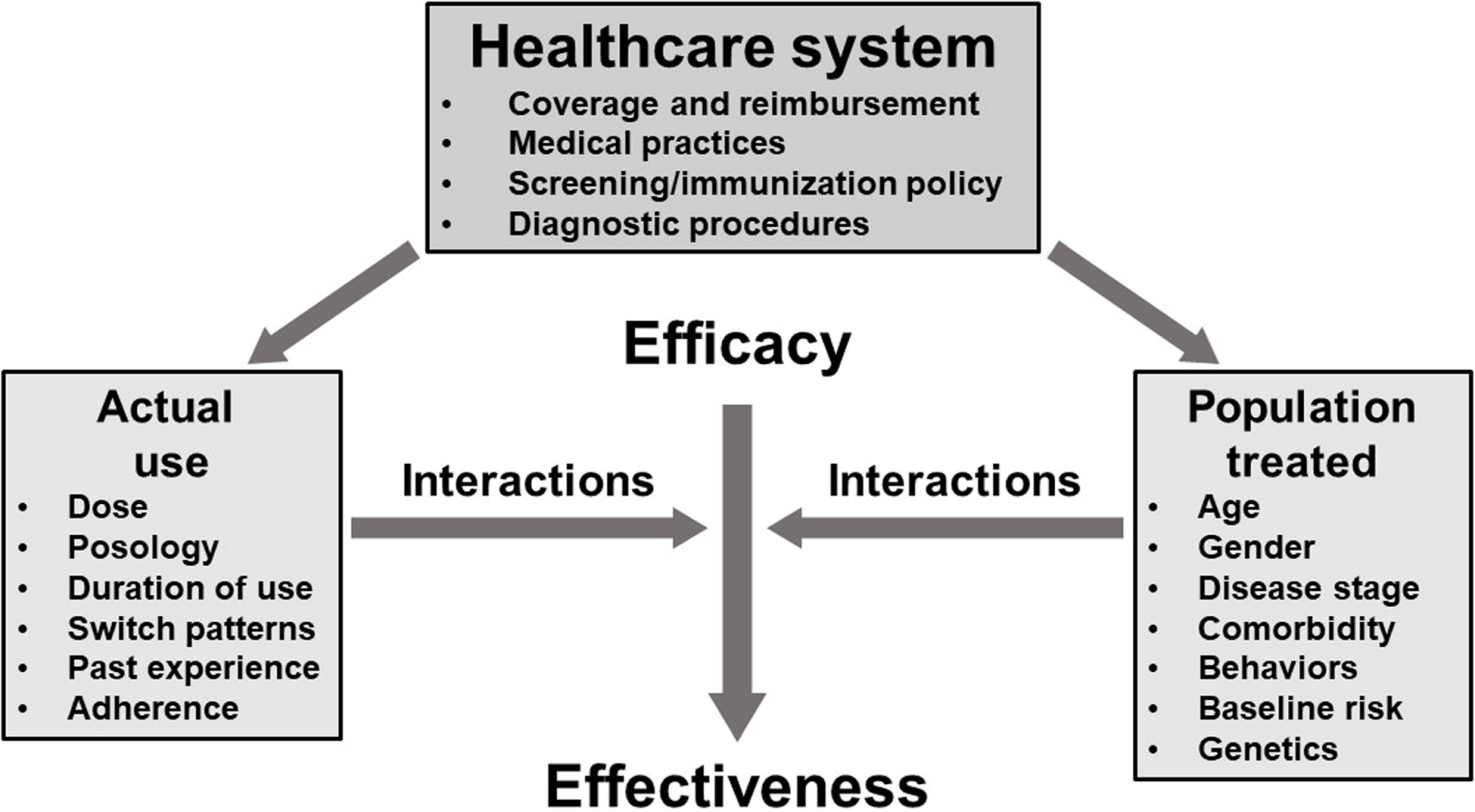
Drug efficacy, factor interactions and effectiveness

A medicine’s efficacy is usually demonstrated under near-ideal conditions in double-blind randomized controlled trials [DBRCTs]) [12]; such trials typically recruit highly selected patient populations and operate under experimental, highly monitored and controlled conditions, which may limit the generalizability of their findings to the broader disease population. Effectiveness can be thought of as the interaction of a medicine’s proven efficacy with factors related to patients, actual medication use, and healthcare systems, which results in the effects observed in patients in the everyday clinical setting (Fig. 1). Abenhaim [13] has described the concept of “drivers of effectiveness” — features of a medicine that may increase or decrease the effectiveness of that medicine in the presence of real-world factors. These drivers of effectiveness encompass a range of factors relating to the patient, the medicine, and the environment, including: (i) patient acceptability, including perceived or real side effects and tolerability; (ii) the medicine’s efficacy; (iii) persistence of correct use of the medicine; (iv) adherence; and (v) affordability, cost-effectiveness and economic factors, e.g. the price the patient may pay for medication and the patient’s age. Other patient-related factors and factors relating to the healthcare system and medical practice, such as vaccination programs, self-management plans in asthma or outreach teams in COPD, may also impact a medicine’s effectiveness and will clearly vary in different healthcare settings.

Abenhaim’s team and the Innovative Medicines Initiative GetReal project have suggested that drivers of effectiveness should be considered early in the drug development cycle [14, 15] and that their impact be explored in appropriately designed studies alongside traditional DBRCTs. As DBRCTs are deliberately designed to remove potential confounders, they are unlikely to allow modifiers of effectiveness to be expressed. It is therefore important, as part of clinical development, that drugs are tested in their intended real-world setting, with minimal intervention (i.e. mimicking everyday clinical practice and preserving the usual behaviors of patients and healthcare professionals as closely as possible) in order to evaluate the medicine’s true effectiveness. The inhaled corticosteroid (ICS)/long-acting β_2_-agonist (LABA) combination, fluticasone furoate/vilanterol (FF/VI [Relvar]; GlaxoSmithKline plc.) was tested in a real-world effectiveness study program. The Salford Lung Studies (SLS) in asthma and COPD evaluated the effectiveness and safety of initiating once-daily inhaled FF/VI versus continuing usual maintenance inhaler therapy (usual care [UC]) in the UK primary care setting. UC comprised a wide variety of inhaled and oral medicines as prescribed by each individual general practitioner (GP) taking part in the study and was not determined by protocol — a major difference compared with typical DBRCTs. The SLS designs and results have been published previously [16–20]. These open-label, pragmatic, randomized, controlled effectiveness trials demonstrated the benefits of initiating FF/VI versus continuing UC in terms of their respective primary endpoints of improvements in asthma control and reduction in COPD exacerbations [19, 20]. The studies were designed to enable GPs to function as study investigators, with changes in treatment during the study permitted based on their clinical opinions.

The results of the SLS raise the questions of what features were driving the improved effectiveness observed for FF/VI versus UC, and how could those drivers of effectiveness help to address some of the obstacles for improving care for patients with asthma and COPD?

## Potential drivers of effectiveness in asthma and COPD

**Fig. 2:**
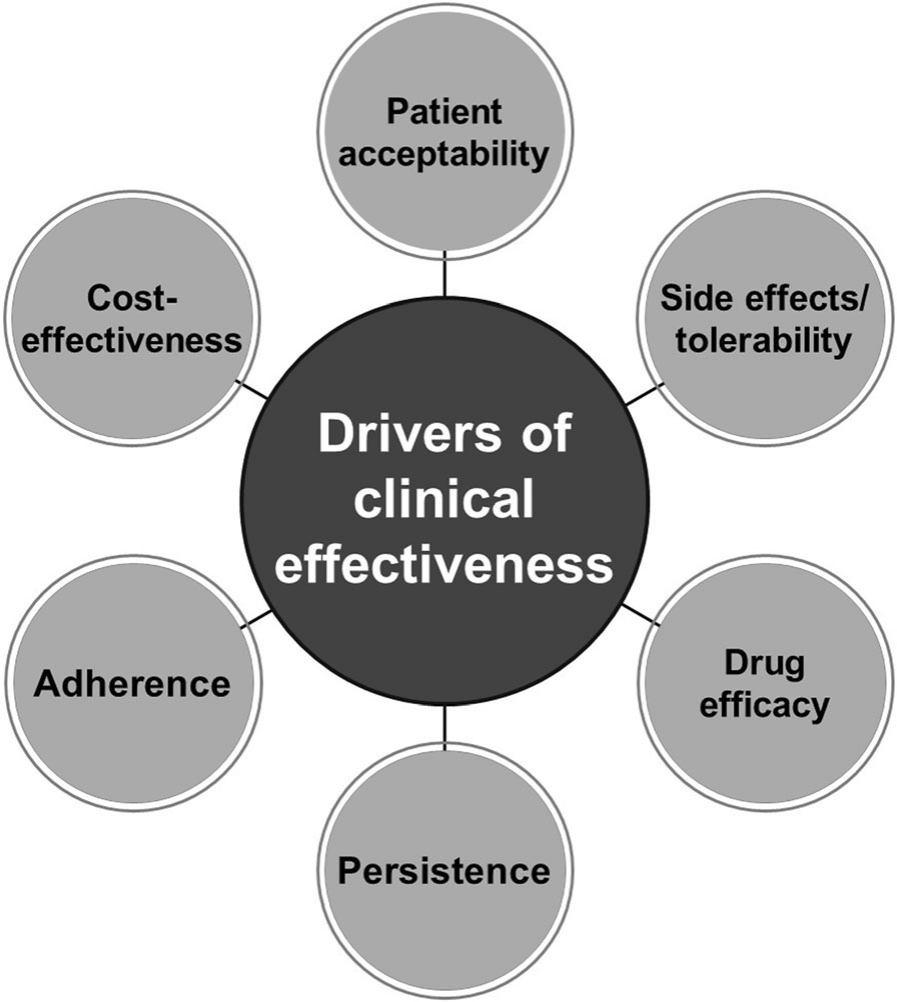
Main drivers of clinical effectiveness

FF/VI delivered via the ELLIPTA dry powder inhaler was designed as an improvement over fluticasone propionate/salmeterol delivered via the Diskus inhaler. An overview of factors thought to be important in driving clinical effectiveness is presented in Fig. 2. Various features of FF/VI could potentially have improved effectiveness and patient outcomes with initiation of FF/VI versus continuation of UC in the SLS, as discussed below.

### Once-daily dosing

Patient adherence with inhaled medications for the treatment of asthma and COPD is low [21, 22] for reasons including patient beliefs, side effects, dosing frequency, and poor inhaler technique [21–23].

In studies of adherence in the real-world setting, adherence rates have been reported to be as low as 10% and typically between 20 and 40% [24–28].

Once-daily treatment administration has the potential to encourage/increase adherence compared with twice-daily administration, as evidenced in medications for asthma and other indications [29–31]. FF/VI was the first once-daily inhaled ICS/LABA combination to be broadly available worldwide. In the SLS, adherence was assessed using the Medication Adherence Report Scale for Asthma (MARS-A) questionnaire and patients’ prescription records were accessed through their electronic case report forms. The MARS-A was used to gather patients’ patterns of medication use (e.g. “I only take it when I need it”), and the number of prescriptions issued was used to estimate the proportion of days covered (PDC) by study medication as a surrogate for treatment adherence. Both methods have their limitations: the MARS-A is a validated questionnaire to assess self-reported adherence, but self-reported behavior does not always reflect actual behavior, such as unintentional non-adherence. Furthermore, the measure captures patients’ general tendencies of how they take their medication, not actual adherence *per se*. The use of prescribing data has considerable limitations in assessing adherence, as it only records the number of prescriptions issued, and not the number dispensed to, or actually used by, patients. Nevertheless, in SLS asthma, the reported mean PDC was 82.3% for FF/VI and 78.2% for UC and in SLS COPD was 85.0% for FF/VI and 82.4% for UC [32, 33]. As planned, no statistical testing has been conducted on these data. Further assessment of adherence to FF/VI through electronic monitoring devices will aid better understanding of this driver of effectiveness [34–37].

### Rapid onset and long duration of action of the active molecules

The rapid onset of action of a medication may result in a perceived benefit to the patient that may encourage treatment adherence [38]. A longer duration of action beyond the licensed dosing interval may mean that the medicine is more “forgiving” of the non-adherence commonly encountered in everyday practice (including irregular dosing and use) [39, 40]. The onset and duration of action of FF/VI has been assessed in asthma. Studies evaluating the bronchodilator effects of FF/VI using serial lung function measures in asthmatic patients have demonstrated an onset of action as early as 15 min [41] and a 72-h duration of bronchodilation after a single dose [42]; slower in onset than formoterol (within minutes [43, 44]) and longer in duration of action than formoterol or salmeterol (at least 12 h) [43–45]. Bardsley et al. examined the duration of airway anti-inflammatory action of FF/VI by serially measuring fractional exhaled nitric oxide (FeNO) over a 14-day treatment period with FF/VI and over 21 days following cessation of therapy. Full suppression of FeNO in asthma was estimated to last for up to 3 days, with effective suppression continuing for up to 18 days, and improvements in forced expiratory volume in 1 s and peak expiratory flow lasting for 3–4 days after cessation of treatment [46]. While there are limited comparative data on the duration of anti-inflammatory action for ICS, separate studies in patients treated with budesonide have reported FeNO return to baseline values within 7 days of cessation of treatment [47].

### Device features and design

Effective drug delivery systems enable the controlled introduction of a medicine into the body, while also improving drug efficacy and safety [48]. The dosage form and device can directly impact on treatment success and patient adherence [48]. Critical errors — those that can be defined as errors resulting in limited or no medication being delivered to the lung — have been associated with major impacts on respiratory symptoms and healthcare consumption [49, 50]. The ELLIPTA inhaler has been shown to be superior to other commonly used inhalers for the administration of ICS/LABA medicines, in terms of patient preference for its design features of dose counter, ease of use, and dosing regimen [51]. Furthermore, it has been shown that fewer patients make critical errors with the ELLIPTA inhaler compared with a range of other ICS/LABA inhalers, and that the ELLIPTA inhaler requires less teaching time than other inhalers [52]. In studies evaluating the dose delivery achieved through ELLIPTA, patients received a dose close to the label claim with inspiratory flow rates of 30 L/min and above 30 L/min peak inspiratory flow rate. Furthermore, studies have shown that asthma and COPD patients across a range of disease severities achieved a flow of 43 L/min or above [53]. In everyday practice, a simple inhaler that requires less time to teach the correct technique, is easy to use, has a low potential for patients to make critical errors, delivers adequate dose across a broad range of inspiratory flow rates, and is preferred by patients, will be a positive driver of effectiveness since there will be greater confidence that the medication has been optimally delivered.

### Tolerability

A theoretical consequence of some drivers of effectiveness is that, while the likelihood of correct and adequate dosing increases, the benefits in terms of positive outcomes might be outweighed by an increased risk of side effects. Tolerability and adverse events reported in phase lll clinical studies of FF/VI in patients with asthma and COPD were similar to those seen with the fluticasone propionate/salmeterol combination [54–56]. In the SLS, serious adverse event rates were very similar for FF/VI and UC [19, 20]. Modeling studies have suggested that FF may have a better therapeutic index than other inhaled steroids [57].

## Discussion

Asthma and COPD guidelines and regulatory and payer frameworks have long favored DBRCTs as constituting the highest level of evidence [3, 58]. Although Cochrane highlighted the importance of understanding the effectiveness of medicines back in 1972 [12], his enthusiasm has not been broadly shared. Pragmatic real-world study designs have not been universally adopted and drug development has instead continued to focus on evaluating efficacy within highly controlled trials in highly selected patient populations. As a result, we are left struggling to assess the external validity of the results of such studies and medicine development programs. As well designed effectiveness studies are undervalued due to their pragmatic design features, the overriding focus on efficacy evaluation is likely to have hampered the implementation of drivers of effectiveness early in drug development processes.

The SLS were world-first, pragmatic, randomized, controlled trials conducted in the routine UK clinical practice setting to evaluate a pre-licensed inhaled medicine [16]. The trials were open-label to maintain their pragmatic design; however, this meant open-label for patients, GPs, pharmacists, other healthcare providers, and most of the study team. This could have introduced bias, particularly as FF/VI would have been either unlicensed or newly licensed while the studies were ongoing. In an attempt to minimise this bias, sponsor study team members who were involved in the development of the analysis plan and the actual data analyses were blinded to patients’ individual therapies up until the formal unblinding of the studies, which occurred after the databases had been finalized. The SLS exemplify that by designing drivers of effectiveness into a medicine, the medicine alone can improve patient outcomes compared to other medications in the same drug class.

It is difficult to assess which components of the composite drivers of effectiveness play the biggest part in improving patient outcomes. Moreover, these drivers are likely to reinforce one another, whereby the physical features of the medicine are improving outcomes and, thus, patient-perceived benefits, which in turn may enhance the belief that the medicine is making a difference. For example, a longer duration of action of a medicine is likely to mitigate any sub-optimal adherence, thus altering the impact of the latter on actual and perceived symptom control. Likewise, an easy-to-use inhaler would enhance the likelihood that the medicine is inhaled correctly, which would increase its effectiveness, as measured and as perceived by patients. We suggest that further work in this field should be pursued for guiding drug developers to design better medicines. We also suggest that regulators, guideline writers, and payers should seek to understand the now well-established concept of effectiveness and build it into their frameworks.

Traditional DBRCTs are deliberately designed to remove potential confounders such as device and patient preference, and thus are unlikely to allow modifiers of effectiveness to be expressed. Such trials rely on highly selected patient populations chosen for their compliance with treatment and study visits, who are typically socially stable, and have high adherence and near-perfect inhaler technique; these patients are not representative of patients seen in everyday clinical practice. Trials such as the SLS show that patients in primary care, recruited with minimal exclusion criteria, can participate in a randomized controlled trial and yield data that complement the data obtained in traditional efficacy DBRCTs.

Currently, we may be ignoring a crucial aspect of medicine assessment and, therefore, denying patients the opportunity for more effective therapies, while also discouraging effectiveness and patient-focused medicine development.

## Conclusions

Evidence suggests that it is possible to design medicines to include a composite of features that can drive effectiveness. Improving a medicine’s effectiveness can provide a meaningful impact on patient outcomes, which can be demonstrated through appropriately designed pragmatic clinical trials. It is time to reconsider evidence hierarchies and bring more external validity to them. This is ultimately likely to benefit patients through encouraging patient-focused drug development, which includes consideration of the drivers of effectiveness and making more effective medicines available to patients.

## Data Availability

Anonymized individual participant data from this study plus the annotated case report form, protocol, reporting and analysis plan, data set specifications, raw dataset, analysis-ready dataset, and clinical study report are available for research proposals approved by an independent review committee. Proposals should be submitted to www.clinicalstudydatarequest.com. A data access agreement will be required.

## Abbreviations

COPD: Chronic obstructive pulmonary disease
DBRCTs: Double-blind randomized controlled trials
FeNO: Fractional exhaled nitric oxide
FF/VI: Fluticasone furoate/vilanterol
GP: General practitioner
ICS: Inhaled corticosteroid
LABA: Long-acting β_2_-agonist
MARS-A: Medication Adherence Report Scale for Asthma
PDC: Proportion of days covered
SLS: Salford Lung Studies
UC: Usual care
